# Prestressed Unbonded Reinforcement System with Multiple CFRP Plates for Fatigue Strengthening of Steel Members ^†^

**DOI:** 10.3390/polym10030264

**Published:** 2018-03-04

**Authors:** Ardalan Hosseini, Elyas Ghafoori, Masoud Motavalli, Alain Nussbaumer, Xiao-Ling Zhao, Roland Koller

**Affiliations:** 1 Swiss Federal Laboratories for Materials Science and Technology (Empa), Überlandstrasse 129, 8600 Dübendorf, Switzerland, and Resilient Steel Structures Laboratory (RESSLab), Swiss Federal Institute of Technology Lausanne (EPFL), Station 18, 1015 Lausanne, Switzerland; 2 Swiss Federal Laboratories for Materials Science and Technology (Empa), Überlandstrasse 129, 8600 Dübendorf, Switzerland; elyas.ghafoori@empa.ch (E.G.); roland.koller@empa.ch (R.K.); 3 Swiss Federal Laboratories for Materials Science and Technology (Empa), Überlandstrasse 129, 8600 Dübendorf, Switzerland, and Department of Civil Engineering, Monash University, Melbourne, VIC 3800, Australia; masoud.motavalli@empa.ch; 4 Resilient Steel Structures Laboratory (RESSLab), Swiss Federal Institute of Technology Lausanne (EPFL), Station 18, 1015 Lausanne, Switzerland; alain.nussbaumer@epfl.ch; 5 Department of Civil Engineering, Monash University, Melbourne, VIC 3800, Australia; zhao.xiao.ling@monash.edu

**Keywords:** steel structures, fatigue crack, fatigue strengthening, carbon fiber reinforced polymer, prestressed unbonded reinforcement, mechanical clamp, finite element simulation

## Abstract

Carbon fiber reinforced polymer (CFRP) composites have exhibited a great potential for strengthening of steel structures. In the current study, an innovative prestressed unbonded reinforcement (PUR) system is introduced for fatigue strengthening of existing steel members. The system relies on a pair of mechanical clamps; each holds multiple CFRP plates and anchors their prestressing forces to the steel substrate via friction. A finite element model was established to optimize the design of the required mechanical components of the system. A set of static and fatigue tests was conducted on the developed mechanical clamps as the key elements of the proposed PUR system. The performance of the PUR system was then evaluated using a set of fatigue tests on two precracked steel plate specimens, one without any strengthening system and the other one strengthened with the proposed PUR system. In the latter specimen, the CFRP plates were prestressed up to about 800 MPa (approximately 30% of the CFRP tensile strength), which resulted in a complete fatigue crack arrest in the precracked steel plate. Furthermore, neither slippage of the mechanical clamps nor any prestress loss in the CFRP plates was observed after 7.5 million fatigue cycles. Based on the promising experimental results, obtained from the sets of fatigue tests performed in the current study, it can be concluded that the proposed PUR system can be considered as an efficient alternative to the conventional bonded reinforcement solutions for fatigue strengthening of damaged steel members.

## 1. Introduction

Fatigue behavior of metallic members has been intensively studied in the past [1], and the efficiency of a number of conventional techniques for the strengthening of fatigue-prone structures (such as drilling stop holes and/or application of bolted, riveted, or welded cover plates) have been experimentally investigated in the literature [2]. Due to the certain advantages of carbon fiber reinforced polymer (CFRP) composites, several pioneer researchers investigated the potential of such advanced materials for the fatigue strengthening of existing metallic structures [3,4,5,6,7]. Adhesively bonding of CFRP reinforcements is considered to be an easy and practical solution for such strengthening applications. Thus, a number of researchers have investigated the bond behavior and debonding capacity of CFRP-to-steel [8,9,10,11], and a few others took into account the effects of near-crack debonding of CFRP from a steel substrate [12,13,14,15]. From a practical point of view, the first known field application of CFRP composites for strengthening of a metallic structure goes back to 2000, when Lane and Ward [16] used wet layup CFRPs for strengthening of Tickford Bridge in the UK. In 2001, Miller et al. [17] used pultruded CFRP laminates for strengthening of Christina Creek Bridge in the United States; while Luke [18] described the strengthening of two historical bridges, i.e., Hythe Bridge in Oxford and Slattocks Canal Bridge in Rochdale, UK, using CFRP plates.

It is obvious that strengthening of fatigue-prone or fatigue-damaged metallic members with prestressed or post-tensioned CFRP composites is more advantageous, due to the fact that a reduction in the tensile stress level that causes damage to the member would be possible by prestressed reinforcements [19,20]. Although the available experimental and numerical studies have demonstrated the aforementioned advantage of prestressed CFRPs [3,19,20,21,22,23,24], fewer attempts have been made to use prestressed bonded reinforcement (PBR) in practical cases [25]. The reason is attributed to the fact that a bonded CFRP reinforcement can carry a limited tensile force before debonding failure occurs [8]. This limited bond capacity, however, can be significantly reduced by prestressing the reinforcement [26], leading to the undesirable premature debonding of the prestressed CFRP reinforcement from the steel substrate under transient service loads.

A series of laboratory tests was performed to compare the behavior of steel beams that were strengthened by PBR and prestressed unbonded reinforcement (PUR) systems [27]. The results have shown that when metallic beams are strengthened by prestressed CFRP plates, the performance of the CFRP-strengthened steel beams is more sensitive to the magnitude of prestress level, rather than the presence of the bond [28,29]. Based on these results, a novel prestressed unbonded CFRP reinforcement system was developed [19] and tested in the laboratory [21]. The system was used for fatigue strengthening of Münchenstein Bridge, a 120-year-old metallic railway bridge in Switzerland [20]. Different shapes and configurations (i.e., trapezoidal, flat, and triangular) of the prestressed unbonded CFRP reinforcement system were suggested in [30] for the strengthening of metallic I-beams. The development and testing of the PUR systems, however, has been so far limited to steel I-beams. Consequently, there is a need for such retrofit system that can be used for the strengthening of existing tensile steel members.

In the current study, an innovative PUR system has been developed as an alternative to the conventional PBR technique for the strengthening of fatigue-damaged steel plates. The system consists of two sets of high performance mechanical clamps, which hold multiple prestressed CFRP plates, and transfer their prestressing forces to the damaged/cracked steel substrate to reduce the acting stress level in the member, and consequently, enhance its fatigue performance. A finite element (FE) simulation is carried out to optimize the required mechanical components of the system, while the important design aspects are discussed. The ultimate capacity and fatigue performance of the developed PUR system are evaluated using a set of static and fatigue tests, which were performed on the proposed mechanical clamping system. Furthermore, the great performance of the developed system in terms of stress reduction in a CFRP-strengthened steel member was demonstrated through a set of fatigue tests on precracked steel plate specimens, with and without the proposed PUR system.

## 2. Finite Element Simulation

### 2.1. Model Description

**Figure 1:**
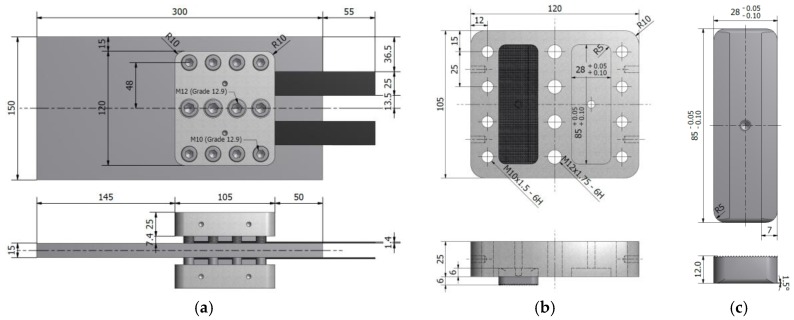
(**a**) Dimensions used in finite element simulation; (**b**) detailed sketch of clamp plate; and, (**c**) detailed sketch of hard plate (all dimensions in mm; (**a**–**c**) not to same scale).

As illustrated in Figure 1a, the proposed mechanical clamping system was designed to hold multiple prestressed CFRP plates on both sides of a steel plate, and transfer their prestressing forces to the steel substrate via friction. The steel plate represents a fatigue-damaged steel member that needs to be strengthened with prestressed CFRP reinforcements. Each set of the mechanical clamping system consists of four toothed hard plates, which press the CFRP plates against the steel substrate using a compression force. This force is generated in the clamp plates by fastening a set of M10 and M12 bolts. In total, eight M10 and four M12 high-strength (grade 12.9) bolts are used in each of the mechanical clamping sets, which are tightened with torques of 54 and 160 Nm, respectively, to generate a total compressive clamping force of 518 kN per clamp set. The detailed sketches of the clamp plate and the hard plate are depicted in Figure 1b,c, respectively.

Given the complexity of the system, a finite element (FE) model was created using Abaqus [31] to optimize the dimensions of the different mechanical components, and to predict the ultimate capacity of the CFRP-steel mechanical joint before slippage of the CFRP plates. The geometry and dimensions used in the FE model are provided in Figure 1a. Owing to the symmetry of the system, however, only one fourth of the system shown in Figure 1a was modeled. The FE model consisted of a CFRP plate with cross-sectional dimensions of 25 × 1.4 mm (width × thickness) that was pressed against the steel substrate via the mechanical clamp. All of the steel parts and the CFRP plate were modelled as isotropic linear-elastic materials with elastic moduli of 209 and 160 GPa, respectively, and a Poisson’s ratio of 0.3.

A hard contact was considered between the clamp plate, hard plate, CFRP reinforcement, and the steel substrate in the normal direction. On the other hand, using the penalty formulation, an isotropic tangential friction with a friction coefficient (*μ_s_*) of 0.4 was introduced between the CFRP–steel substrate, as well as between the CFRP–hard plate. The steel substrate was modelled using 8-node linear brick elements of type C3D8R with reduced integration and hourglass control, while CFRP plate was modelled using 20-node quadratic brick elements of type C3D20R with reduced integration. Both the hard plate and the clamp plate were modelled using a 10-node quadratic tetrahedron of type C3D10HS with improved surface stress visualization. The elements had an average dimension of approximately 2 mm, giving a total number of approximately 89,500 elements for the entire model.

**Figure 2:**
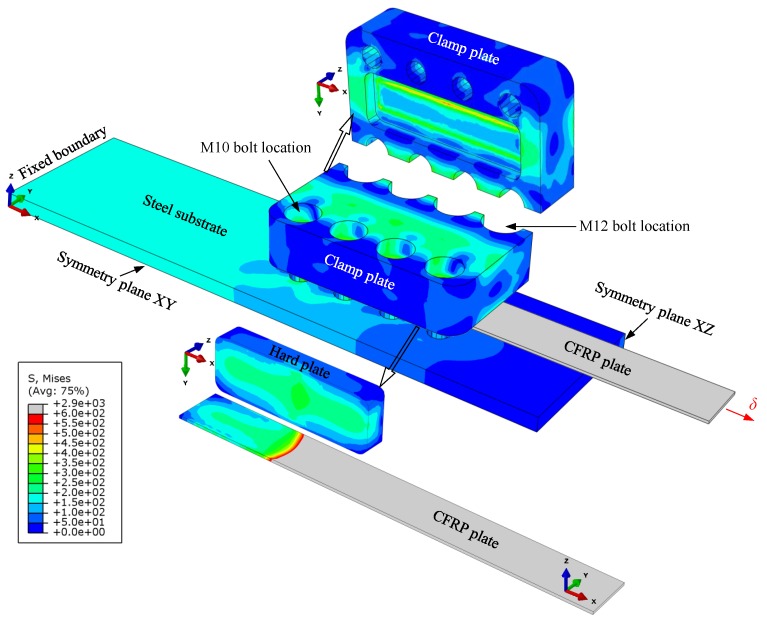
Finite element model created in Abaqus and distribution of von Mises’ stresses in different components of the proposed mechanical clamping system at ultimate anchorage capacity.

In addition to the symmetry boundary conditions, a fixed support boundary was introduced at the steel plate extremity to stabilize the model assembly (see Figure 2). In the first loading step in Abaqus, a uniform pressure of 0.001 MPa was applied on the clamp plate to activate the defined contact interactions between different components. It is important to note that avoiding this loading step can pose a convergency problem. In the second loading step, static uniform pressures of 218 and 396 MPa per bolt location were introduced on the clamp plate to simulate the prestressing forces of 28.8 and 72 kN per bolt, which was generated in M10 and M12 high-strength bolts, upon fastening with the allowable torques of 54 and 160 Nm, respectively. In the final Abaqus loading step, a uniform displacement-controlled loading was applied to the free edge of the CFRP plate to evaluate the anchorage capacity of the joint before slippage of the mechanical clamping system.

### 2.2. Finite Element Results

Figure 2 shows the distribution of von Mises’ stresses in different components of the FE model, when the full bolts preload has been applied on the clamp plate, and the CFRP-steel joint was loaded up to its ultimate capacity. It can be seen from Figure 2 that by using 25 mm-thick clamp plates, manufactured from M200 steel with a nominal yield strength (*σ_y_*) of 1000 MPa, the maximum stress in the clamp plate is below 0.6*σ_y_*. This criterion was initially considered to design the thickness of the clamp plate. Furthermore, Figure 2 illustrates that an almost uniform contact pressure was reached between the CFRP plate and the hard plate. This was achieved by cutting 7-mm transverse fillets with an inclination angle of 1.5° on the surface of the hard plate, which is in contact with the clamp plate (see Figure 1c). Note that obtaining an almost uniform contact pressure between the hard plate and the CFRP strip, especially in the transverse direction, is of crucial importance for the proposed system. The reason is attributed to the fact that any stress concentrations on the CFRP edges can result in longitudinal delamination of unidirectional CFRP plates, when prestressed/loaded.

**Figure 3:**
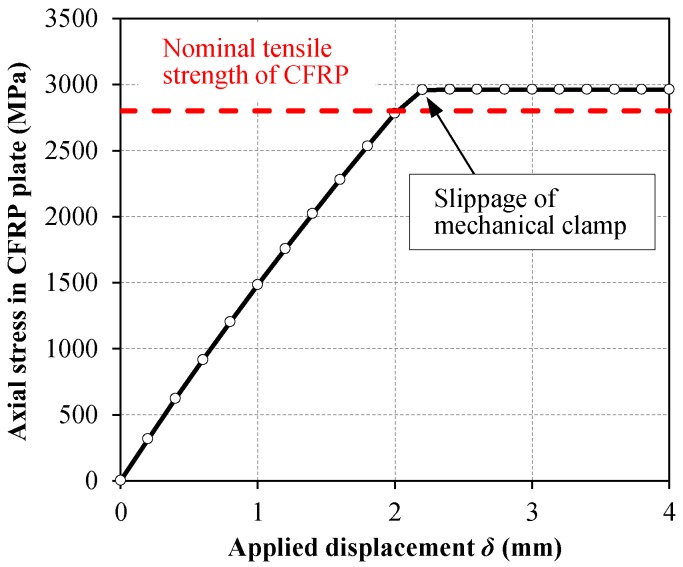
Finite element (FE) results: axial stress vs. applied displacement to carbon fiber reinforced polymer (CFRP) free edge mechanically clamped to steel substrate.

The generated axial stress in the CFRP plate as a function of the applied displacement on the CFRP free edge was obtained from the FE simulation, and the results were used to plot the stress–elongation response of the CFRP plate provided in Figure 3. Based on the obtained FE results, it can be concluded that the proposed mechanical clamping system is capable of carrying the entire tensile strength of the CFRP plate, *σ_f,u_* (a nominal value of 2800 MPa was considered for *σ_f,u_*) before slippage of the clamp.

## 3. Experimental Program

### 3.1. Test Specimens

**Figure 4:**
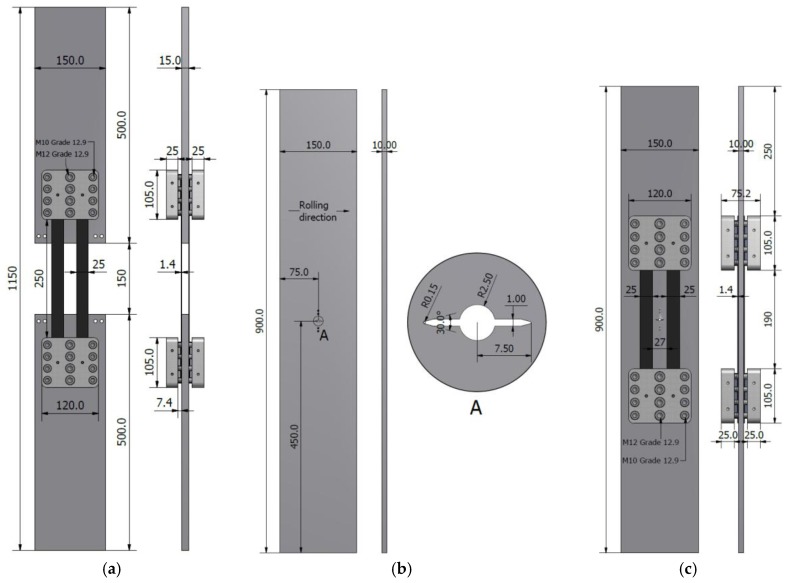
Specimen dimensions (all dimensions are in mm): (**a**) clamp test specimen; (**b**) reference specimen and electrical discharge machine (EDM) notch details; and, (**c**) prestressed unbonded reinforcement (PUR) specimen.

In the current study, two sets of tensile tests were carried out. The first set of experiments was conducted on so called clamp test specimens, as depicted in Figure 4a, to evaluate the ultimate capacity and fatigue performance of the proposed mechanical clamping system, as the main component of the PUR system. As it is shown in Figure 4a, the clamp test specimen was specially designed to evaluate the capacity of the proposed mechanical clamping system for anchoring CFRP plates to steel substrate via friction.

In the second set of the experiments, the performance of the PUR system was investigated by conducting fatigue tests on two precracked middle-tension (M(T)) steel plate specimens, one without any strengthening as the reference specimen (Figure 4b), and the other one strengthened with the proposed PUR system (Figure 4c). Further details regarding the test specimens that were used in the second set of the experiments can be found in [32].

### 3.2. Prestressed Unbonded Reinforcement (PUR) System

#### 3.2.1. Mechanical Clamps

**Figure 5:**
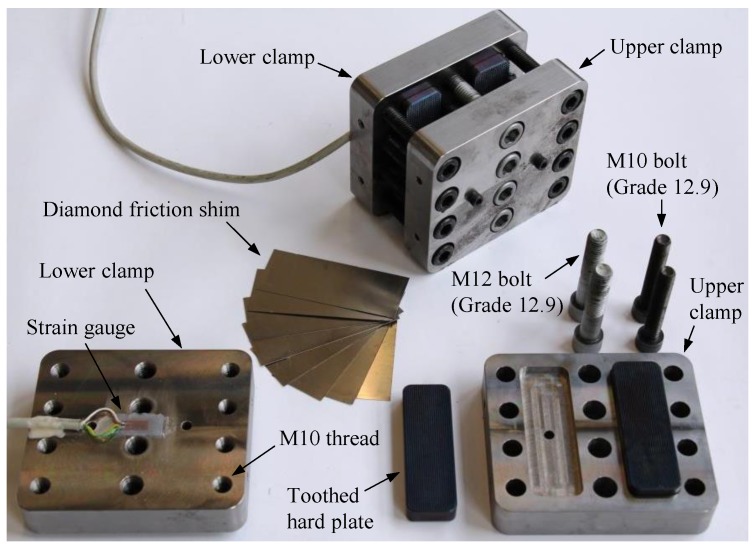
Different components of the developed mechanical clamping system.

Different components of the proposed mechanical clamping system, with the optimized dimensions, are illustrated in Figure 5. As it can be seen in Figure 5, each set of the designed clamps consists of four toothed hard plates with a hardness of HRC 58(-60) on the Rockwell scale. Owing to the fact that the entire clamping system functions with the help of friction, 3M^TM^ diamond friction shims of grade 10 (3M Technical Ceramics GmbH, Kempten, Germany), were used between the CFRP plates and the steel substrate to increase the friction. Furthermore, because the normal force that is generated by the prestressed bolts is transferred to the hard plates via the upper and lower clamp plates and causes relatively high bending stresses in those parts (see Section 2.2), the upper and lower clamp plates were manufactured from high strength steel M200 with a nominal yield strength of 1000 MPa.

It should be mentioned here that the PUR system, which is developed in the current study, was specially designed to hold multiple CFRP plates (two CFRP plates on each side of the steel plate) apart at the crack tips (Figure 4c). This allows for the state of the crack to be visually monitored, which is of great interest in laboratory fatigue experiments, as well as in practical cases. It is obvious that the PUR system can be designed to hold only two CFRP plates (one on each side of the steel member) covering the fatigue-damaged zone. This would lead to a relatively simpler design procedure for the mechanical clamping system with fewer mechanical components being involved. However, proportionally lower prestressing forces can be achieved when fewer CFRP plates are used.

#### 3.2.2. Prestressing Setup

**Figure 6:**
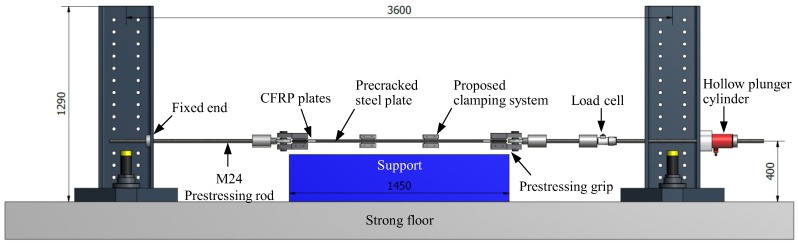
Prestressing setup (all dimensions are in mm).

To strengthen the precracked specimen (Figure 4c) in the second phase of the experiments, a prestressing setup (Figure 6) was designed and assembled at the Structural Engineering Research Laboratory of Empa in order to simultaneously prestress four parallel CFRP plates and anchor their prestressing forces to the precracked steel plate using the developed mechanical clamping system. To do so, the four CFRP plates were first placed in the specially designed prestressing grips (see Figure 6). Using a hydraulic hollow plunger cylinder, an average prestrain level of 5180 μm/m (approximately 30% of the nominal strength of the composite) was generated in the CFRP plates.

The prestrain level was obtained based on an analytical calculation of the mode I stress intensity factor (SIF) range (Δ*K_I_*) in the PUR specimen, to be less than an assumed mode I threshold SIF range Δ*K_I,th_* (considering Δ*K_I,th_* = 100 N/mm^3/2^) to obtain complete crack arrest (see [32] for the comprehensive background and further details). The aforementioned prestrain value in the CFRP plates corresponded to a load level of 110.8 kN, which was monitored using a 300 kN load cell along with all of the strain gauges on the CFRP plates and the steel specimen upon prestress force release. Immediately after prestressing the CFRP plates, the high-strength bolts of the mechanical clamps were tightened with the required torque using a digital torque meter. The prestressing force in the cylinder was then released to zero and the CFRP plates were cut from both sides of the mechanical clamps to realize the final configuration, as depicted in Figure 4c.

### 3.3. Material Properties

With the exception of the mechanical components of the proposed clamping system, which were manufactured from high strength M200 steel with a nominal yield strength of 1000 MPa, the utilized steel plates in all the experiments were of type S355J2+N with a nominal yield strength of 355 MPa. Elastic modulus, yield, and ultimate strength of the utilized steel along the rolling direction were obtained to be 205 GPa, and 421 and 526 MPa, respectively, while no significant difference in the mechanical properties of the steel parallel or perpendicular to the rolling direction was observed. Further details regarding the auxiliary tensile tests that were performed to characterize the mechanical properties of the utilized steel can be found in [32]. Furthermore, NM CFRP plates of type S&P 150/2000 with measured cross-sectional dimensions of 25 × 1.4 mm (width × thickness) were used. The nominal tensile strength of the CFRP plate is 2800 MPa based on the manufacturer’s catalogue, while the elastic modulus was measured as 156 GPa [33].

### 3.4. Static and Fatigue Test Setup

**Figure 7:**
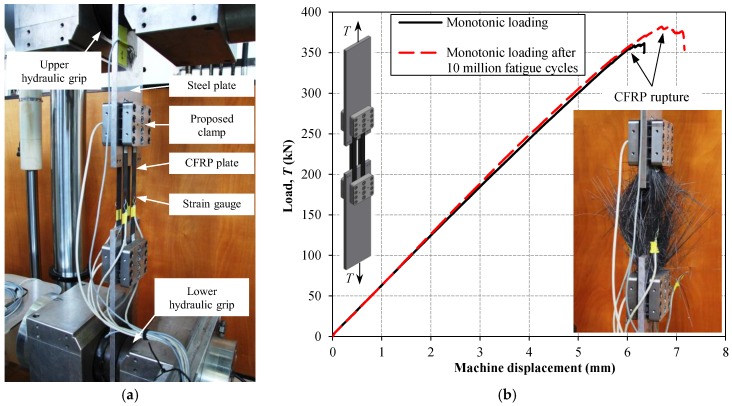
(**a**) Static and fatigue test setup; and (**b**) load–displacement response of clamp test specimens and CFRP rupture in monotonic loading after 10 million fatigue cycles.

A 1-MN static/fatigue servo-hydraulic Schenck machine with an Instron controller was used to perform static and fatigue tensile tests on the clamp test specimens (i.e., the first phase of the experiments), as well as fatigue tests on the reference and PUR specimens (i.e., the second phase of the experiments). Static tensile tests on the clamp test specimens (Figure 4a) were performed under displacement-control conditions at a speed of 1 mm/min, while the fatigue test on this specimen configuration was performed under the load-control condition with a load ratio (*T_min_*/*T_max_*) of *R* = 0.9, and a frequency of 18 Hz. Figure 7a shows the test setup and the instrumentation (i.e., electrical foil strain gauges of type 1–LY61–6/120 and 1–LY66–6/120 mounted on steel and CFRP plates, respectively) used to monitor the clamp test specimens during static and fatigue loading.

In the second phase of the experiments, fatigue tests were performed on the reference and PUR specimens (Figure 4b,c) under the load-control condition with a load ratio of *R* = 0.2 and a frequency of 15 Hz. It is worth mentioning here that the selected load ratio of *R* = 0.2, in this case was deemed to represent the practical load ratio that was experienced by most of the fatigue-prone members in metallic bridges. Assuming *R* = 0.2 for a metallic member (to be strengthened), the load ratio experienced by the PUR system can be calculated as *R* = 0.9 using the stress state in the prestressed CFRP reinforcements [33]. Thus, in case of fatigue tests, performed in the first and second phases of the experiments, *R* = 0.9 and 0.2 were used, respectively.

## 4. Results and Discussions

### 4.1. Static and Fatigue Tests on the Proposed Mechanical Clamping System

Figure 7b illustrates the load–displacement response of the two clamp test specimens, which were monotonically loaded until failure. First, a clamp test specimen was placed in the testing machine (Figure 7a) and the ultimate capacity of the proposed mechanical clamping system was evaluated using a monotonic tensile loading. It can be seen from Figure 7b that no reduction in the joint stiffness neither any slippage of the clamping system was observed until tensile rupture of the CFRP plates occurred (see Figure 7b). The second clamp test specimen was first subjected to 10 million fatigue cycles. The maximum load level during the fatigue test was equal to *T_max_* = 162.5 kN, which corresponded to 41% of the nominal tensile strength of the utilized CFRP. Afterwards, a monotonic load was applied on the specimen until the ultimate strength of the CFRP plates was reached. The experimental results provided in Figure 7b demonstrate that the proposed mechanical clamps are capable of transferring the entire tensile capacity of the CFRP plates to the steel substrate, even after experiencing 10 million fatigue cycles. Careful inspection of Figure 7b reveals that the second clamp test specimen (subjected to 10 million fatigue cycles) exhibited slightly higher stiffness when compared to the first specimen especially at higher tensile load levels. This is believed to be the influence of fatigue loading, as cyclic loading can align the individual fibers within the composite with respect to the tensile loading direction, and, consequently, a slightly higher stiffness and ultimate strength of the CFRP reinforcement can be achieved.

**Figure 8:**
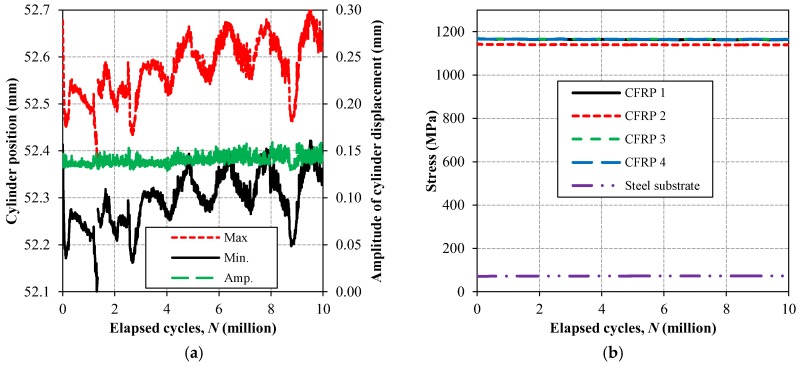
(**a**) Evolution of cylinder position during fatigue test; and, (**b**) evolution of maximum stress in CFRP plates and steel substrate in response to fatigue cycles.

Evolution of the maximum and minimum cylinder position, as well as the amplitude of the cylinder displacement are illustrated in Figure 8a for the fatigue test performed on the second clamp test specimen. It can be seen from the experimental results of Figure 8a that both of the curves, representing maximum and minimum of the cylinder position, experienced considerable fluctuations over fatigue cycles. This is deemed to be mainly due to the temperature difference during the fatigue testing period (approximately 6.5 days). However, the amplitude of the cylinder displacement is almost constant over the elapsed 10 million fatigue cycles, which strongly proves the fact that the proposed mechanical clamping system experienced no slippage during the elapsed 10 million fatigue cycles. Moreover, the evolution of the maximum stress in the four CFRP plates, as well as the steel substrate with respect to fatigue cycles is provided in Figure 8b. It can be seen from the experimental results of Figure 8b that the maximum stress levels in the four CFRP plates were almost identical, and these levels of stress in the CFRPs and the steel substrate were quite constant over the 10 million fatigue cycles. The aforementioned observation firstly verifies that all the CFRP plates were uniformly loaded, and secondly, none of the four CFRP plates slipped out from the proposed mechanical clamping system during the fatigue test. It is worth mentioning that the stress values, provided in Figure 8b, are calculated by multiplying the elastic modulus of CFRP and steel by the corresponding strain values, obtained from the mounted electrical strain gauges on CFRPs and steel (Figure 7a), which were monitored during the fatigue test using a digital data acquisition system.

### 4.2. Performance of the Proposed PUR System for Fatigue Strengthening of Cracked Steel Members

**Figure 9:**
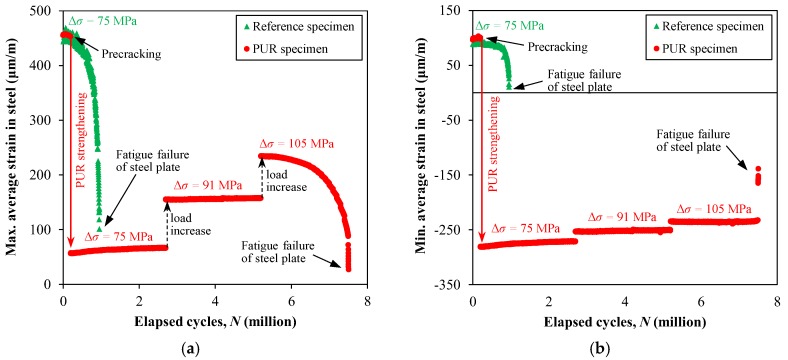
Evolution of: (**a**) maximum; and (**b**) minimum average strain in steel with respect to fatigue cycles (Δ*σ* = fatigue stress range applied to the unstrengthened cross section of steel).

**Figure 10:**
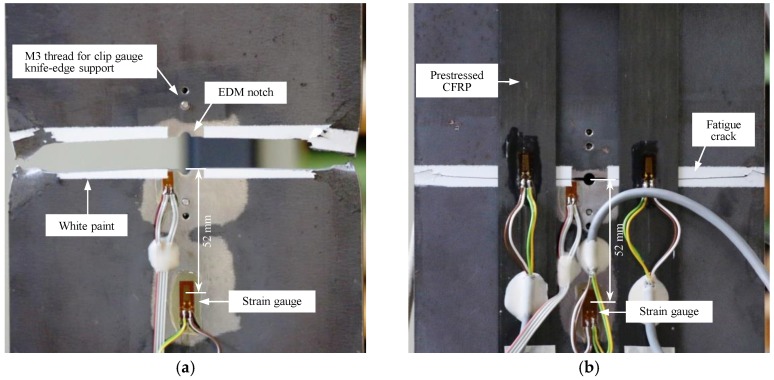
Arrangement of strain gauges and fatigue failure in (**a**) reference specimen; and (**b**) PUR specimen.

The evolutions of the maximum and minimum average steel strain (i.e., average of strain readings on both sides of the steel plate) in the reference and PUR specimens with respect to the fatigue cycles are plotted in Figure 9. It can be seen in the figure that the maximum and minimum average strain level in the reference specimen, subjected to the fatigue stress range (Δ*σ*) of 75 MPa, gradually reduced by increasing the number of elapsed fatigue cycles. The reason is attributed to the fact that, the strain gauges used to monitor the strain level in the steel plates were mounted at the mid-width of both sides of the steel plates at a distance of 52 mm from the specimens’ mid-length (see Figure 10). Consequently, the fatigue crack growth resulted in a gradual reduction in the strain readings until the complete fatigue failure of the reference specimen occurred at *N* = 0.935 million cycles (see Figure 10a).

Figure 9 shows that applying the PUR system on the precracked steel plate used in the PUR specimen considerably reduced the strain level in the steel substrate. It can be seen from the figure that, during the fatigue cycles, the maximum and the minimum strain levels in the steel substrate remained constant for the first two 2.5 million cycles, with a sudden increase at 2.7 million cycles due to the 20% increase in Δ*σ*. This proves that the proposed PUR system experienced no slippage during fatigue loading as no reduction in the prestressing effect was observed. On increasing Δ*σ* from 91 to 105 MPa after 5.2 million cycles, a second sudden increase in the steel strain levels was observed, owing to the increase in the applied loading. Similar to the reference specimen, the strain level in the steel substrate was then started to decrease with the number of cycles because of the fatigue crack re-initiation and propagation. A sudden jump in the strain levels was observed when the crack finally propagated through the entire steel section (see Figure 10b).

The experimental results provided in Figure 9 demonstrates that, the strengthening of the cracked steel plate using the proposed PUR system considerably reduced the tensile portion of the acting cyclic stresses. This reduction in the tensile stresses then resulted in Δ*K_I_* values to be less than Δ*K_I,th_* for Δ*σ* = 75 and 91 MPa. Increasing Δ*σ* by 40% to 105 MPa, however, re-initiated the fatigue crack, and resulted in a gradual reduction of measured strain values in the steel. It should be noted here that, the main intention of discussing the fatigue test results on the reference and PUR specimens in this section was to show the superior performance of the proposed PUR system for fatigue strengthening of cracked steel members. Thus, a detailed discussion on the fracture mechanics aspects of crack propagation and crack arrest in the reference and PUR specimens is beyond the scope of the current paper, and readers may refer to [32] for a comprehensive discussion on the aforementioned topics.

## 5. Summary and Conclusions

In the current study, an innovative friction-based mechanical clamping system was introduced to strengthen fatigue-prone or fatigue-damaged steel members using multiple prestressed unbonded CFRP plates. First, an FE simulation was carried out to optimize the design of the required mechanical components of the proposed mechanical clamping system, and the important design aspects were discussed. The ultimate capacity of the mechanical clamping system was then predicted using the established FE model. Afterwards, a set of static and fatigue tests was performed to evaluate the ultimate capacity and fatigue performance of the proposed mechanical clamping system. Experimental results strongly confirmed that the developed mechanical clamping system is capable of transferring the entire tensile capacity of the CFRP plates to the steel substrate, even after experiencing 10 million fatigue cycles. The performance of the developed PUR system for fatigue strengthening of cracked steel members was then evaluated in a set of fatigue tests on precracked steel plate specimens. Experimental results showed that the existing fatigue crack in the precracked steel plate, strengthened with the proposed PUR system, was completely arrested even for the 20% higher stress range, when compared to the initial stress range applied on the reference specimen. Furthermore, neither slippage of the mechanical clamps nor any prestress loss in the four CFRP plates was observed during 7.5 million fatigue cycles. Consequently, owing to the advantages of the developed PUR system, such as the capability of applying relatively high prestressing forces to arrest an existing fatigue crack without the need for any surface preparation and curing process, which are often required for bonded solutions, the proposed system can be considered as a good alternative to the conventional bonded CFRP reinforcements for fatigue strengthening of damaged steel members.

## Nomenclature

CFRP	Carbon fiber reinforced polymer	*N*	Number of fatigue cycles (-)
EDM	Electrical discharge machine	*R*	Fatigue loading ratio (-)
NM	Normal modulus	*T_max_*	Maximum tensile fatigue load (kN)
PBR	Prestressed bonded reinforcement	*T_min_*	Minimum tensile fatigue load (kN)
PUR	Prestressed unbonded reinforcement	*μ_s_*	Friction coefficient (-)
SIF	Stress intensity factor	Δ*σ*	Fatigue stress range (MPa)
Δ*K_I_*	Mode I SIF range (N/mm^3/2^)	*σ_f,u_*	CFRP tensile strength (MPa)
Δ*K_I,th_*	Mode I threshold SIF range (N/mm^3/2^)	*σ_y_*	Steel yield strength (MPa)
